# Curiosities of Ancient Leechcraft

**Published:** 1887-05-14

**Authors:** 


### CURIOSITIES OF ANCIENT LEECHCRAFT.

BY A STUDENT OF OLD BOOKS AND MANUSCRIPTS.

(*Continued from p.* 98, *vol. ii.*)

EARTHWORMS pickled in vinegar, green lizards boiled alive in oil, scorpions, centipedes, powdered unicorns' horns (no information is given as to how these were to be obtained), serpents' skins steeped in vinegar—such are some of the popular drugs of the antique pharmacœia. Human saliva is recommended for many complaints, both external and internal; and perspiration was such a favourite therapeutic agent that the owners of Greek gymnasia were wont to scrape it from the bodies of athletes and sell it. This, when mingled with oil, was regarded as having "emollient, calorific, and expletive" qualities. Nervousness was to be cured by a diet of cooked vipers. There is an appearance of reason in this prescription. Anyone who was possessed of strength of will sufficient to overcome his natural revulsion from such fare, might well be supposed to gain by the effort the power to control his nerves in any possible human emergency.

The nursery belief, that if a thing is disagreeable it must be good for you, seems to have ruled the minds of the old doctors; and we are not surprised to learn that the Athenian physicians of the classic age were in the habit of taking a rhetorician to accompany them on their rounds, and trusting to his oratory to persuade their patients into swallowing the nauseous medicines they prescribed.

The medical atrocities of early times, which have been already given, might be considered sufficiently shocking, but still more odious examples remain to be mentioned. We read with pity, tempered with disgust, of the devoted Chinese daughter who chops off one of her fingers in order that her mother may regain health by partaking of a broth made from it. But her sacrifice, the benefit of which is obtained by direct favour from the gods, and not from any supposed medical quality in the flesh itself, is more deserving of sympathy, however repulsive it may seem, than the cannibalism recommended by the doctors of old. The ghastly idea that human flesh, medically prepared, was a specific in various diseases doubtless arose in very early times, and originated in the custom of offering human sacrifices to Odin and other gods. It is believed that the bodies of these victims, or parts of them, were eaten by the chiefs, not as a meal but as a sacramental observance. The Aztecs, for example, used every spring to sacrifice a young man, his heart being solemnly eaten by the nobles of the tribe.

But looking at such practices, as we do, as horrors of which only barbarous nations would be guilty, it is rather surprising to learn that a modification of this odious custom obtained in England in such recent and civilised times as the reign of Charles II. It will naturally be assumed that at least it was only by ignorant charlatans that cannibalism was recommended; not by a noted physician, the Jenner of his day. Nevertheless, it is from the work of no less a personage than Dr. Edward Bolnest, Physician in Ordinary to the King, that we propose to quote. In 1672 this gentleman published and dedicated to the Duke of Buckingham a treatise on therapeutics, on the title-page of which he declares his purpose of giving a "Rational way of preparing animals, vegetables, and minerals for a physical use, by which they are made most efficacious, safe, and pleasant medicines for the preservation and restoration of the life of man."

From this volume we give the method of preparing what the author styles a "mummiall quintessence":—

"Take of the flesh of a sound young man dying a natural death about the middle of August, three or four pounds. Put it into a fit glass, and pour upon it spirit of wine. Let it stand so three or four days. Take out the flesh and put it upon a glass plate, and imbibe it with spirit of salt. Let it stand uncovered, but in the shade where no dust or other filth may fall upon it. Be sure you often turn, and being well dried you may put it up in a fit jar and keep it fit for use."

The period of the year when the victim to medicine dies is always made an important point of, as is the conjunction of stars which witnesses his death. If, for example, the "sound young man" did not postpone his departure from this world till August, but died in spring, he could not, with due regard to therapeutic propriety, be mummified; but he was not, therefore, useless to medicine. His blood, carefully distilled, could be made into a balsam, which Dr. Bolnest vouches for as being "a potent preservative in time of pestilence, and in leprosie, palsie and gout of all sorts," if it is "taken in broth or treacle-water with a fasting stomach." No wonder the people of those days believed in ghosts. Without being exceptionally superstitious, one may well fancy that a man who had breakfasted on broth flavoured with human blood, might be haunted by the original owner of that blood, who would, in all honesty, have the right to impair his digestion of the ghastly meal.

After such a gruesome dose the "Remedy for Arthritick Pains," consisting of the calcined bones of a man "which hath not been buried fully a year," is hardly worth mentioning; and one can regard with perfect satisfation the depilatory action of earth taken out of a human skull, and the tonic qualities of plants which have grown in such a flower-pot.

Sad to say, however, Dr. Bolnest did not find his medicines as reliable as he desired; for though he declares that in time of need patients may "in some measure" trust his prescriptions, he admits sadly, "I never yet from the best of medicines always found those certain effects I could have desired." Indeed, considering the strange ideas the worthy man had of his science, it is remarkable, if Dr. Bolnest attended Charles II on his deathbed, that the monarch had not cause to express surprise as well as contrition that he took "such an unconscionable time in dying."

There were innumerable cures (?) which were as revolting to the physical nature of the patient as those we have cited must have been to his moral instincts. Does it not seem an act of positive cruelty to add to the fever and pain of small-pox by hanging the patient's room with scarlet, wrapping him in scarlet, giving him nothing but scarlet on which to cast his eyes? The idea is maddening! Yet many sufferers underwent this treatment. Some even survived it. After this method went out of fashion it was customary to hire nurses (fat ones preferred) to lie by the patient's side and keep him warm. As such a service was well paid, there were not wanting many poor women who were ready to undertake it.

And if such was the medicine of those days, what shall be said of its surgery! When as yet anæsthetics were not, when the lightest experiment must have been attended with an agony which we, who like to inhale nitrous oxide gas when we have a tooth extracted, shudder to contemplate, the knife and the red-hot iron were used with appalling recklessness. We can only hope that nerves were not so sensitive then as now. Blisters were considered good for almost every disease, and amputations, of a good slashing kind, with no precautions for tying up arteries and the like, were readily undertaken. The limb was sawn off, and a hot-iron was applied to the place to staunch the bleeding. If the patient died of fear before, or of agony during the operation, it was considered in no way remarkable; and public opinion, which now through the medium of the press chronicles in large letters and severely criticises a rare death under chloroform, then took the accident as a matter of course.

In a milder form this readiness to shed human blood survived to our own times. It is not certain that even now there are no old-fashioned practitioners who look upon bleeding as a wholesome remedy for fevers; though we trust there are none who would approve the treatment applied by a Florentine doctor to a young mother entrusted to his care. This unfortunate lady was bled from the arm nineteen times during the fortnight after the birth of her child and before—but it is scarcely necessary to add—her untimely death.

Such, then, was ancient medicine; but though we have spoken of the folly and barbarity that characterised much of it, it must be remembered that alongside this blundering and cruelty physiological knowledge was increasing, and valuable discoveries were being made. While rash and conceited practitioners were nauseating their hapless patients, careful and diligent students of disease were doing work which forms the foundation of modern medical science.

